# Low Pretreatment Albumin-to-Globulin Ratios Predict Poor Survival Outcomes in Patients with Head and Neck Cancer: A Systematic Review and Meta-analysis

**DOI:** 10.7150/jca.80955

**Published:** 2023-01-09

**Authors:** Yun-Ting Wang, Liang-Tseng Kuo, Chia-Hsuan Lai, Yuan-Hsiung Tsai, Yi-Chan Lee, Cheng-Ming Hsu, Chun-Ta Liao, Chung-Jan Kang, Ethan I. Huang, Ming-Shao Tsai, Geng-He Chang, Yao-Te Tsai

**Affiliations:** 1 Department of Otorhinolaryngology-Head and Neck Surgery, Chang Gung Memorial Hospital, Chiayi, Taiwan.; 2 College of Medicine, Chang Gung University, Taoyuan, Taiwan.; 3 Division of Sports Medicine, Department of Orthopedic Surgery, Chang Gung Memorial Hospital, Chiayi, Taiwan.; 4 Department of Radiation Oncology, Chang Gung Memorial Hospital, Chiayi, Taiwan.; 5 Department of Diagnostic Radiology, Chang Gung Memorial Hospital, Chiayi, Taiwan.; 6 Department of Otorhinolaryngology-Head and Neck Surgery, Chang Gung Memorial Hospital, Keelung, Taiwan.; 7 Department of Otorhinolaryngology-Head and Neck Surgery, Chang Gung Memorial Hospital, Taoyuan, Taiwan.

**Keywords:** albumin-to-globulin ratio, head and neck cancer, meta-analysis, survival outcomes, biomarker

## Abstract

**Background:** Studies have indicated that a low albumin-to-globulin ratio (AGR) before treatment is linked to poor prognosis of many cancers, but the prognostic impact of AGR remains controversial in head and neck cancer (HNC). This meta-analysis examined the prognostic value of AGR in HNC.

**Methods:** We systematically searched the Embase, PubMed, and Cochrane library for relevant articles from inception to July 22, 2022. Studies conducted from 2000 to 2022 exploring the prognostic value of AGR in HNC were retrieved. We employed a random-effects model and calculated pooled hazard ratios (HRs) with corresponding 95% confidence intervals (CIs) to examine the associations of AGR with survival outcome.

**Results:** Our analysis included nine studies involving 3211 patients with HNC. The pooled results revealed significant associations between low pretreatment AGRs and poor disease-free survival (HR = 1.97, 95% CI 1.58-2.45, p < 0.001), distant metastasis-free survival (HR = 1.64, 95% CI 1.25-2.16, p < 0.001), overall survival (HR = 2.18, 95% CI 1.65-2.88, p < 0.001), T3-T4 status (OR = 2.22, 95% CI 1.43-3.44, p < 0.001), stage III-IV disease (OR = 2.62, 95% CI 1.62-4.23, p < 0.001), and lymph node metastasis (OR = 1.95, 95% CI 1.29-2.82, p = 0.001) in patients with HNC.

**Conclusion:** AGR can serve as a prognostic biomarker in managing HNC, and a low pretreatment AGR is strongly associated with adverse survival outcomes and advanced cancer status. Additional large-scale prospective trials must be conducted to assess the validity of our findings.

## Introduction

Head and neck cancers (HNCs), including tumors that originate from the epithelium of the nasopharynx, larynx, hypopharynx, oropharynx, and oral cavity, are heterogeneous 1. HNCs are the sixth most common malignancy worldwide and result in more than 200,000 deaths annually 2. Although the prognosis of HNCs has improved, the 5-year survival rate is approximately 60% and varies significantly among patients with tumors at different locations 1. The main reasons for treatment failure in HNCs are locoregional recurrence and distant metastasis 3, and the high risk of second primary cancers in head and neck regions create a considerable therapeutic challenge 4. The tumor-node-metastasis (TNM) staging system is commonly used for HNC prognostics and treatment planning. However, the TNM system accounts only for anatomical tumour extension, and the survival outcomes of patients with the same disease stage often vary. Therefore, the identification of effective biomarkers that facilitate the early identification of patients with poor prognosis and the development of personalised treatment plans are crucial.

Because host nutrition and systemic inflammatory responses play major roles in tumorigenesis, disease progression, and cancer management 5, 6, numerous studies have explored the impacts of these factors on the prognosis of patients with cancer. Humans' main serum protein components, albumin (ALB) and globulin (GLB), have essential functions in systemic inflammatory responses and ligand transportation 7. Researchers have identified ALB and GLB levels and the albumin-to-globulin ratio (AGR) as valuable prognostic markers in patients with various cancers, including colorectal cancer 8, brain tumor 9, urothelial cancer 10, and gastric and lung cancer 11, 12. Given that malnutrition is frequently observed in patients with HNC before treatment 13, studies have investigated the relationship between pretreatment AGR and survival in patients with HNC 3, 14, 15. However, because of the single-institution nature of these studies with small sample sizes as well as variations in study design, the results of these studies have remained inconclusive. A meta-analysis including 24 studies and 13,890 patients with solid tumours indicated that a high AGR was linked to better survival in patients with all disease stages and tumor locations, except for patients with oesophageal cancer 16. However, this meta-analysis included only two studies on nasopharyngeal carcinoma (NPC), and to date, there is no quantitative and comprehensive assessment on the prognostic role of AGR in HNC. Therefore, we conducted this systematic review and meta-analysis to verify whether the AGR can predict prognosis and clinicopathological features of HNC, so as to render more cogent evidence to support its use in HNC management. Because AGRs can reflect both host nutrition status and systemic inflammatory responses, we hypothesised that AGR would be significantly associated with HNC prognosis.

## Materials and Methods

### Search strategy

We conducted the systematic review according to the Preferred Reporting Items for Systematic Reviews and Meta-analyses reporting guidelines 17, and the study was registered in PROSPERO (CRD42022365813). Because the collected data were obtained from published articles, approval from an institutional review board was not necessary. Studies that included patients with HNC and investigated the prognostic value of AGR were eligible for inclusion. We conducted a search for eligible studies in Embase, Medline, PubMed, and the Cochrane Central Register of Controlled Trials from inception to July 22, 2022. We also searched the registry of trials administered by the US National Institutes of Health (http://clinicaltrials.gov) for ongoing clinical trials. Details regarding the search strategy and keywords used to search each database are listed in the Supplementary Materials (Table S1). The reference lists of the selected studies were independently and manually screened for potentially relevant publications by two review authors (Y.T.W. and Y.T.T.). We did not apply restrictions related to publication language, publication year, or participant ethnicity.

### Study selection

To be eligible for inclusion in our review, articles were required to (1) report studies including patients with pathological diagnoses of HNC, (2) report studies investigating the association between AGR and survival outcomes, and (3) report relevant hazard ratios (HRs) and 95% confidence intervals (CIs) or sufficient information for their calculation. We excluded (1) case reports, letters, conference abstracts, reviews, and opinions; (2) articles that provided insufficient data for survival analysis; and (3) articles reporting nonhuman trials. The studies identified through the searches were independently evaluated by two authors (Y.T.W. and Y.T.T.). First, the titles and abstracts of all the selected articles were screened, and irrelevant studies were excluded. Thereafter, the full texts of the remaining articles were reviewed, and were screened texts for eligibility according to exclusion and inclusion criteria. Any inconsistencies were resolved through consultation with the third author (L.T.K.).

### Data extraction

The two authors who conducted the search (Y.T.W. and Y.T.T.) independently extracted the following information from the included articles: (1) study information (year of publication, study region, study design, name of the first author, sample size, and follow-up period); (2) patient clinicopathological characteristics (cancer stage, tumour location, T status, lymph node metastasis, cancer cell differentiation, and treatment modality); (3) AGR cutoff values and the methods used to determine them; and (4) HRs with 95% CIs for survival analysis, including those for overall survival (OS), local relapse-free survival (LRFS), distant metastasis-free survival (DMFS), disease-free survival (DFS), and locoregional recurrence-free survival (LRRFS). The HRs and 95% CIs were extracted from the articles or were obtained from the survival curves and raw data through the use of methods proposed by Parmar and Tierney 18, 19. If both univariate and multivariate data were provided, the adjusted HRs were used for a pooled analysis. The third author (L.T.K.) checked the extracted data.

### Quality assessment

The two authors who extracted the data (Y.T.W. and Y.T.T.) used the Newcastle-Ottawa quality assessment scale (NOS) 20 to independently evaluate the methodological quality of the included studies. Scores on the NOS range from 0 to 9 points; we considered studies with scores of >6 to have high methodological quality. Any disagreements regarding the scores were resolved through consultation with the third author (L.T.K.).

### Statistical analysis

Comprehensive Meta-Analysis software (version 3; Biostat, Englewood, NJ, USA), was applied to conduct all statistical analyses, and a p value of <0.05 represented statistical significance. To account for potential heterogeneity among the included studies, we employed a random-effects model 21. We used Cochran's *Q* test and *I*^2^ values to measure heterogeneity among the included studies; a p value of <0.10 was considered statistically significant. *I*^2^ values of 75%-100%, 50%-74%, 25-49.9%, and 0%-24.9% indicated high heterogeneity, moderate heterogeneity, low heterogeneity, and no heterogeneity, respectively 22. If significant heterogeneity was detected, we performed subgroup analyses to explore factors that may influence the prognostic value of AGR in HNC. We also performed sensitivity analysis involving sequential removal of each study to examine the robustness of the pooled results. We calculated odds ratios (ORs) and corresponding 95% CIs to measure the associations between AGR and clinicopathological characteristics, and a funnel plot test was performed to identify potential publication bias 23.

## Results

### Search results

**Figure 1:**
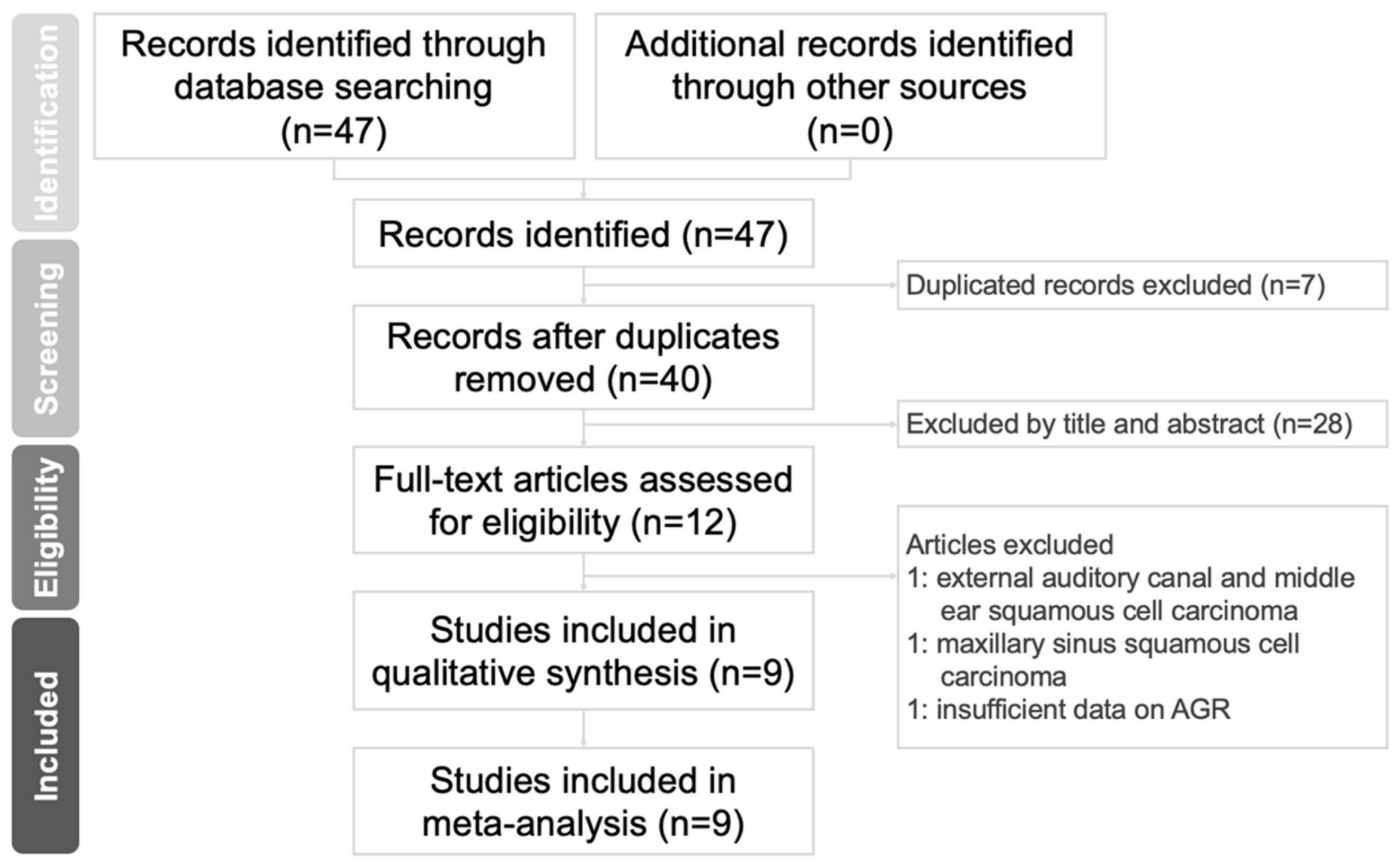
Flowchart of study selection.

A flowchart of the study selection procedure is presented in Figure 1. We retrieved 47 relevant published articles through our initial search. After excluding seven duplicates, we excluded 28 more articles after the abstracts and titles were screened. After the full texts of the remaining 12 articles were screened, three records were excluded; the reasons for exclusion are specified in Figure 1. Ultimately, nine studies involving 3211 patients with HNC 3, 14, 15, 24-29 met the eligibility criteria and were entered in our meta-analysis.

### Study characteristics

The characteristics of enrolled studies are summarised in Table 1. All the included studies were retrospective and published between 2014 and 2021. The sample sizes ranged from 76 to 792 (median, 255). All the studies included patients with a stage I-IV disease; four, three, and two included patients with NPC 3, 25-27, oral cavity cancer 14, 24, 29, and laryngeal cancer 15, 28, respectively. The AGR cutoff values ranged from 1.19 to 1.6 (median, 1.34) and were determined using receiver operating characteristics curve analysis in six studies 3, 14, 15, 25, 26, 28, X-tile software in two studies 24, 29, and a method proposed by Igarashi et al. 30 in one study 27. Only two of the selected studies investigated the association between AGR and LRFS/LRRFS; therefore, OS 3, 14, 15, 24, 25, 27-29, DFS 14, 15, 25, 28, 29, and DMFS 3, 26, 27 were selected as the primary outcomes of interest in our meta-analysis. All the included studies had NOS scores of >6 points (Table S2).

### Association of AGR with OS

**Figure 2:**
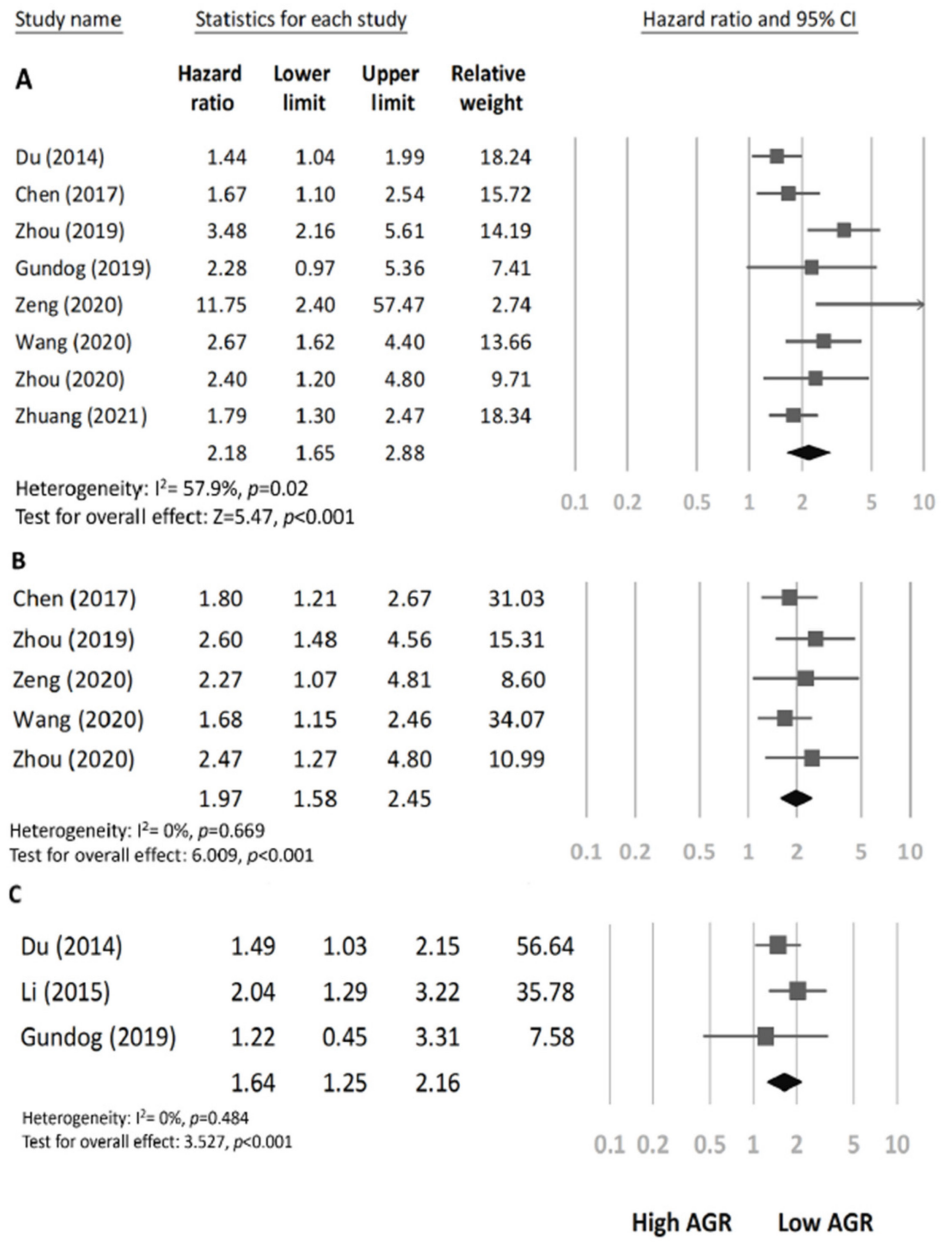
Associations between AGR and survival outcomes. **A** Forest plot of the association between overall survival and AGR in patients with HNC. **B** Forest plot of the association between disease-free survival and AGR in patients with HNC. **C** Forest plot of the association between distant metastasis-free survival and AGR in patients with HNC.

We detected moderate heterogeneity among the eight studies (involving 2691 patients) reporting a link between AGR and OS (I^2^ = 57.9%; P_H_ = 0.02). According to the pooled results, a low AGR before treatment is linked with poor OS in HNC (pooled HR 2.18, CI 1.65-2.88, p < 0.001; Figure 2A).

In our search for sources of heterogeneity, we performed subgroup analyses stratified by tumor location (nasopharynx, oral cavity, or larynx), sample size (≤255 or >255), AGR cutoff value (≤1.34 or >1.34), and the method used to determine the cutoff value (ROC or others; Table 2). The results indicated that a low AGR had a significant association with poor OS in patients with laryngeal cancer (pooled HR=2.38, 95%CI 1.29-4.40, p = 0.006), oral cavity cancer (pooled HR=2.21, 95%CI 1.31-3.71, p = 0.003), or NPC (pooled HR=2.14, 95%CI 1.15-3.98, *p* = 0.016). Furthermore, the heterogeneity among subgroups stratified by tumor location varied, suggesting that tumor location may have caused the observed heterogeneity. Regarding sample size, AGR cutoff values, and methods used to determine the cutoff values, AGR had a significant association with OS in all the subgroups, suggesting that our results were both robust and reliable.

### Association of AGR with DFS/DMFS

The pooled HR from the five studies (involving 1110 patients) that reported an association between AGR and DFS exhibited minimal heterogeneity (*I*^2^ = 0.0%; P_H_ = 0.669; Figure 2B) and indicated that a low AGR could predict poor DFS (pooled HR=1.97, 95%CI 1.58-2.45, p < 0.001). The three studies (involving 1309 patients) that reported an association between AGR and DMFS also exhibited low heterogeneity (I^2^= 0.0%; P_H_ = 0.484; Figure 2C) and demonstrated that a low AGR has a significant association with poor DMFS in HNC (pooled HR=1.64, 95%CI 1.25-2.16, p < 0.001).

### AGR and clinicopathological factors

We analyzed the relationships between AGR and seven clinicopathological variables: gender, age, smoking status, T status, TNM stage, lymph node metastasis, and cancer cell differentiation (Table 3). The results revealed that a low AGR had a significant association with stage III-IV disease (OR=2.62, 95%CI 1.62-4.23, p < 0.001), T3-4 status (OR=2.22, 95%CI 1.43-3.44, p < 0.001), and lymph node metastasis (OR=1.95, 95%CI 1.29-2.82, p = 0.001). No significant associations were observed between AGR and age (OR=1.29, 95%CI 0.91-1.82, p = 0.154), gender (OR=0.76, 95%CI 0.51-1.14, p = 0.182), smoking status (OR=0.84, 95%CI 0.40-1.74, p = 0.63), or cancer cell differentiation (OR=0.85, 95%CI 0.61-1.22, p = 0.38).

### Sensitivity analysis and Publication bias

**Figure 3:**
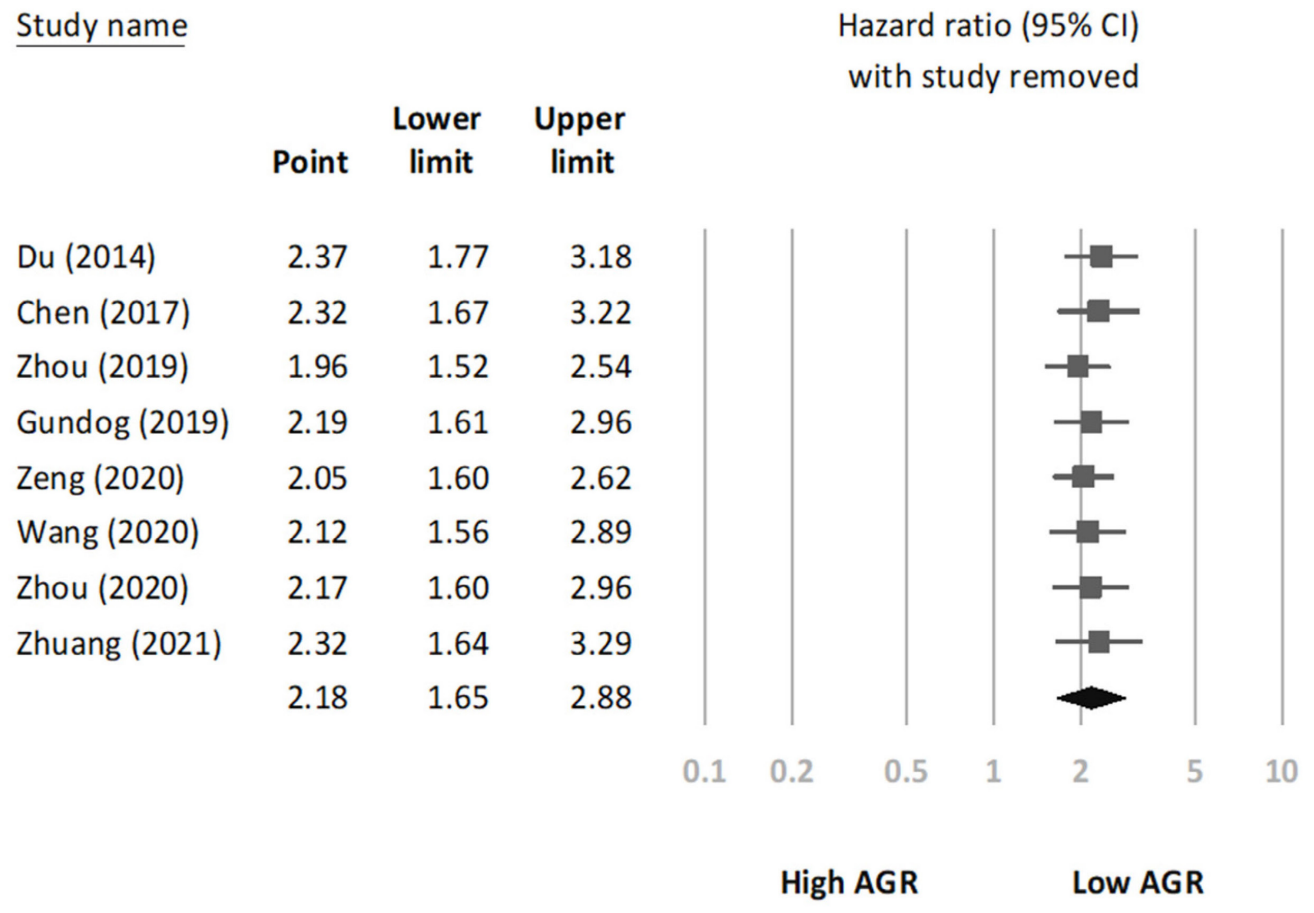
Sensitivity analysis.

**Figure 4:**
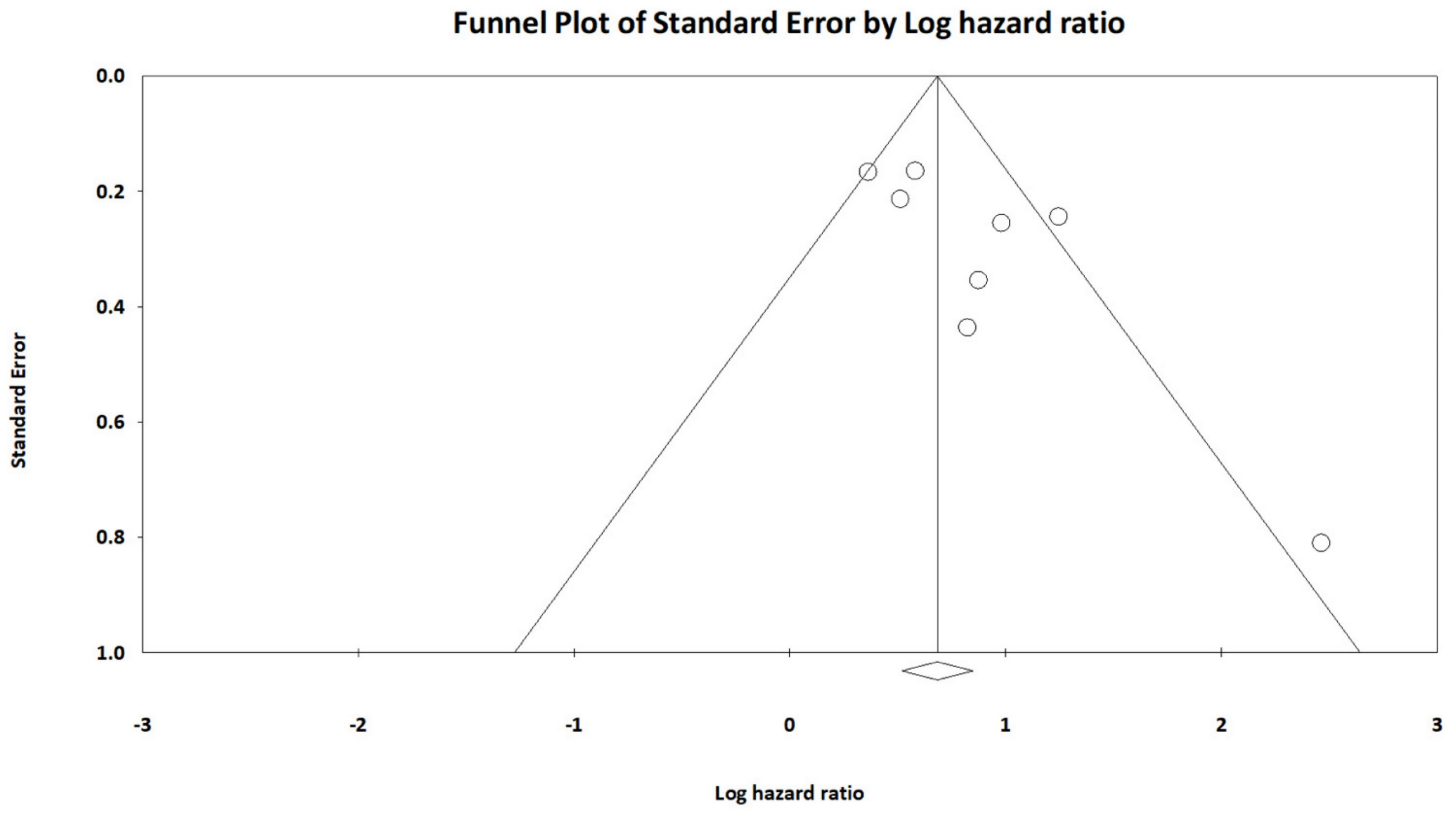
Funnel plot of hazard ratios for overall survival based on AGR.

We conducted a sensitivity analysis to assess the robustness of the association between AGR and OS through the sequential omission of each study from the pooled analysis (Figure 3). The results revealed that the association between AGR and OS did not change significantly, indicating that our findings were reliable. In addition, we constructed a Begg's funnel plot (Figure 4) and detected no significant publication bias (p = 0.22).

## Discussion

According to our literature review, this was the first meta-analysis to examine the utility of the AGR for predicting survival outcomes and investigate the relationships between AGR and clinicopathologic characteristics in HNC. Our analysis included nine studies involving 3211 patients, and the pooled results revealed that a low pretreatment AGR is significantly associated with poor DFS, OS, and DMFS in patients with HNC. Because HNC is a heterogeneous disease, the tumor location may strongly affect the survival of patients with HNC. However, our results demonstrated that AGR had a significant association with OS in patients with tumours originating in the oral cavity, nasopharynx, or larynx, suggesting the common applicability of AGR in HNC prognosis. In addition, our subgroup analyses stratified by sample size, AGR cutoff value, and the method used to determine the AGR cutoff value achieved significant and consistent results. The heterogeneity among the studies involving patients with oral cavity cancer was minimal, but that among the studies involving patients with NPC and laryngeal cancer was high, suggesting that the observed heterogeneity may be attributable to tumor location. Our sensitivity analysis further supported the reliability of our findings. In addition, we discovered that a low pretreatment AGR is associated with advanced pathological characteristics of HNC, such as neck lymph node metastasis and advanced T status, implicating that measuring a patient's AGR before treatment may facilitate the early detection of adverse tumour features. Based on these findings, we suggested that the AGR may be clinically applicable as a cost-effective prognostic marker in the management of HNC, and the patients with HNC and low pretreatment AGRs may benefit from more careful and personalized treatment planning and careful follow-up. To facilitate the use of AGR in clinical practice, several researchers have proposed the use of AGR-based nomograms that integrate AGR and clinicopathological characteristics, which have been demonstrated to accurately predict the OS of patients with HNC 14.

The mechanisms underlying the relationship between AGR and prognosis of HNC remain uncertain but may be related to inflammatory responses and nutritional status, both of which are reflected in a patient's AGR. ALB, which is synthesized by hepatocytes, is the most abundant protein in human serum and is frequently used to assess patients' nutritional statuses. In addition, ALB helps maintain vascular integrity and permeability and serves as a major free radical scavenger in the human body 31. The antioxidant and antitumor effects of ALB help ensure the stability of cell DNA replication and inhibit the proliferation of cancer cells 32. Therefore, patients with low ALB levels are considered to have a poor nutritional status and weakened antitumor immunity. Patients with HNC frequently experienced cachexia and hypoalbuminemia 33; both of which are associated with poor prognosis 34. In addition, serum ALB levels may be correlated with host inflammatory responses 35, which are involved in the tumorigenesis and progression of HNC 36. Aggressive cancer invasion may increase cancer-related inflammation and cytokine production, suppressing the biosynthesis of ALB 37. Various proinflammatory proteins, including immunoglobulins, complement components, and C-reactive protein, fall under the umbrella of GLB 38. The upregulation of some inflammatory cytokines in serum GLBs, such as tumor necrosis factor, IL-6, and IL-8, could promote tumor progression, immune escape, and metastasis 39, which are closely linked to poor prognoses in patients with HNC 40. Low AGR levels may reflect a poor nutritional status, increased inflammatory responses, and weak antitumor immunity, which may contribute to poor prognosis in HNC. However, the mechanisms underlying the associations between AGR and prognosis in HNC warrant further investigation.

Researchers have conducted meta-analyses to explore the associations between AGR and the prognoses of various human malignancies. One meta-analysis of 28 studies involving 15,356 patients with various types of cancer revealed significant associations between a low pretreatment AGR and poor PFS, DFS, and OS 41; however, it only included two studies involving patients with HNC. A meta-analysis focusing on gastric cancer had similar results 42. Our results were also consistent with the aforementioned findings, suggesting that AGR may be used as a prognostic marker in cancer management. In addition, the clinical value of AGRs extends beyond the prediction of survival outcomes. In one large retrospective study involving 26,974 healthy adults, the participants with low AGRs were at a higher risk of various malignancies, including HNC 43. Regarding the associations between AGR and clinicopathological factors, a meta-analysis of 14 studies involving 4136 patients with 11 types of cancer demonstrated that low AGRs were significantly associated with an elevated risk of lymph node metastasis 44. Another meta-analysis of eight cohort studies involving 2668 patients with urologic cancer revealed that lymphovascular invasion and T and N status differed significantly between patients with high and low AGRs 45. In another study involving 306 patients with oral cavity cancer, a low AGR (<1.55) was associated with adverse clinicopathological characteristics, such as advanced cancer stage, extranodal extension, and lymph node metastasis as well as a depth of invasion of ≥10 mm 14. The aforementioned results had similar trend with our findings, which may be attributable to several factors. First, a substantial tumor burden may be associated with cachexia 46 and decreased serum ALB levels 47, resulting in a lower AGR. Moreover, patients with large head and neck tumours have higher serum levels of proinflammatory and proangiogenic cytokines 48, which may lead to a low serum ALB concentration 49 and, in turn, a low AGR. However, the mechanisms underlying the aforementioned associations warrant further investigation.

Although ALB and GLB can be used to predict the clinical outcomes of patients with cancer 50, 51, the measurement of a single indicator may be affected by host factors, such as body fluid changes, hepatic dysfunction, and acute infection or inflammation. Therefore, ALB or GLB levels alone may not serve as an effect predictor of survival outcomes in patients with HNC. However, AGR may be less influenced by variations attributable to measurement methods or the aforementioned individual factors and is therefore a more reliable prognostic marker than ALB or GLB alone. In addition, the sensitivity of AGR in survival prediction is high. Suh et al. conducted a retrospective study involving 26,974 healthy adults and discovered that some individuals with normal total serum protein and albumin levels had low AGRs (<1.1) 43. Analysing patients' AGRs may help health-care providers identify patients at risk of unfavourable survival outcomes who cannot be identified on the basis of their serum ALB levels.

The strength of our meta-analysis is represented by its quantitative and systematic evaluation of the clinical role of AGR in patients with HNC, which is, to our knowledge, the first meta-analysis in this field. The enrollment of large numbers of patients enabled us to verify the prognostic effect of AGR in different tumor locations and cut-off intervals, further increasing the general applicability of AGR in HNC management. Based on the observed associations between a low AGR and adverse pathological characteristics of HNC, our findings suggested that pretreatment AGR may serve as a useful tool for early detection of advanced HNC. As a literature-based meta-analysis, this study has several limitations of note. First, all the included studies employed retrospective designs and may therefore have been subject to bias. Second, most of the included studies involved Asian populations; therefore, our findings may be applicable to Asian populations but not generalisable to individuals of other races. Third, no consensus has been reached regarding the optimal AGR cutoff value for predicting survival in patients with HNC; the resulting heterogeneity among the included studies may limit the applicability of our findings. Furthermore, although most of the HRs were derived from multivariate analyses, different sets of variables may have been included in different studies, which may have influenced the reliability of our results. Because of these limitations, additional large-scale well-designed prospective studies are warranted to assess the validity of our findings and further explore the optimal AGR cutoff value for use in predicting the survival outcomes of patients with HNC.

In conclusion, our study results indicated that a low pretreatment AGR is significantly associated with poor DFS, DMFS, and OS as well as stage III-IV disease and neck lymph node metastasis in patients with HNC. Because measuring a patient's AGR is convenient and simple, AGR may serve as a cost-effective biomarker for prognostics and individualized treatment planning. Researchers should conduct additional randomized and multicentre prospective trials to assess the utility of AGR in clinical practice and the associations between AGR and other clinicopathological biomarkers.

## Supplementary Material

Supplementary tables.

## Figures and Tables

**Table 1:** Characteristics of included studies

First author	Year	Study region	Sample Size	Tumor location	Cancer Stage	Treatment	Cut-off value resource	AGR Cut-off	Survival outcome	HR analysis	Follow-up (months)	NOS
Du	2014	China	694	Nasopharynx	I-IV	RT or CRT	Others*	1.4	OS, DMFS, DFS, LRFS	M	N/A	7
Li	2015	China	520	Nasopharynx	I-IV	RT or CRT	ROC	1.34	DMFS	M	88.4	8
Chen	2017	China	241	Larynx	I-IV	Surgery +/- adjuvant	ROC	1.28	OS, DFS	U	73	8
Gundog	2019	Turkey	95	Nasopharynx	I-IV	RT or CRT	ROC	1.19	OS, LRRFS, DMFS	U	41	8
Zhou	2019	China	232	Larynx	I-IV	Surgery +/- adjuvant	ROC	1.31	OS, DFS	M	27.3	7
Zeng	2020	China	255	Nasopharynx	I-IV	RT or CRT	ROC	1.5	OS, DFS	M	33.5	6
Wang	2020	Taiwan	306	Oral cavity	I-IV	Surgery +/- adjuvant	ROC	1.55	OS, DFS	M	49.2	8
Zhou	2020	China	76	Oral cavity	I-IV	Surgery +/- adjuvant	X-tile software	1.6	OS, DFS	M	48	7
Zhuang	2021	China	792	Oral cavity	I-IV	Surgery +/- adjuvant	X-tile software	1.34	OS	M	27.48	8

*Method proposed by Igarashi et al. 30. Abbreviations: CRT: chemo-radiotherapy; DFS: disease- free survival; DMFS: distant metastasis-free survival; LRFS: local relapse-free survival; LRRFS: loco-regional recurrence-free survival; M: multivariate analysis; N/A: not applicable; NOS: Newcastle- Ottawa scale; OS: overall survival; RT: radiotherapy; ROC: receiving operating characteristics; U: univariate analysis.

**Table 2:** Summary of subgroup analysis results of AGR in OS

Subgroup	No. of studies	No. of patients	Pooled HR (95%CI)	*p* value	Heterogeneity	
					*I*^2^ (%)	*P_h_*
**Overall**	8	2691	2.18 (1.65-2.88)	<0.001	57.9	0.02
**Tumor sites**						
Nasopharynx	3	1044	2.14 (1.15-3.98)	0.016	71.83	0.029
Larynx	2	473	2.38 (1.29-4.40)	0.006	80.49	0.024
Oral cavity	3	1174	2.21 (1.31-3.71)	0.003	0	0.377
**Sample size**						
>255	4	2047	2.05 (1.37-3.05)	<0.001	68.62	0.023
≤255	4	644	2.36 (1.55-3.59)	<0.001	41.64	0.162
**Cut-off of AGR**						
>1.34	4	1331	2.28 (1.43-3.63)	<0.001	70.18	0.018
≤1.34	4	1360	2.16 (1.42-3.28)	<0.001	52.85	0.095
**Methods for determining AGR cutoff**						
ROC	5	1129	2.60(1.84-3.67)	<0.001	56.36	0.057
Others	3	1562	1.73 (1.2-2.49)	0.003	1.24	0.363

Abbreviations: AGR: albumin-to-globulin ratio; ROC: receiving operating characteristics.

**Table 3:** Odds ratio analysis between clinicopathological and AGR

Variables	No. of studies	No. of patients	OR (95%CI)	*p* value	Heterogeneity	
					*I*^2^ (%)	*P_h_*
Age (>60 vs. <60)	3	549	1.29 (0.91-1.82)	0.154	0	0.752
Gender (Male vs. Female)	6	1644	0.76 (0.51-1.14)	0.182	42.39	0.123
Smoking (yes vs. no)	4	644	0.84 (0.40-1.74)	0.63	75.37	0.007
T stage (T3-4 vs. T1-2)	5	1568	2.22 (1.43-3.44)	<0.001	71.53	0.007
Lymph node metastasis (yes vs. no)	4	1243	1.95 (1.29-2.82)	0.001	47.29	0.128
TNM stage (III-IV vs. I-II)	5	1403	2.62 (1.62-4.23)	<0.001	63.92	0.026
Differentiation (P-D vs. W-D/M-D)	4	855	0.85 (0.61-1.22)	0.38	0	0.953

## Abbreviations

AGR: albumin-to-globulin ratio
ALB: albumin
CI: confidence interval
DFS: disease-free survival
DMFS: distant metastasis-free survival
GLB: globulin
HNC: head and neck cancer
HR: hazard ratio
LRFS: local relapse-free survival
LRRFS: locoregional recurrence-free survival
NOS: Newcastle-Ottawa quality assessment scale
NPC: nasopharyngeal carcinoma
OR: odds ratio
OS: overall survival
TNM: tumor-node-metastasis
