# Supplementary material for: Low Pretreatment Albumin-to-Globulin Ratios Predict Poor Survival Outcomes in Patients with Head and Neck Cancer: A Systematic Review and Meta-analysis
Source: J Cancer. 2023 Jan 9;14(2):281–9. doi: 10.7150/jca.80955 (PMC9891875; doi:10.7150/jca.80955)
Supplement: Supplementary file 1 — Supplementary tables. [file jcav14p0281s1.pdf]

Table S1. Search strategies.

**CENTRAL (via Cochrane Register of Studies)**	**PUBMED**	**EMBASE (Ovid)**
#1 MeSH descriptor: [Head and Neck Neoplasms] explode all trees<br><br>#2 MeSH descriptor: [Otorhinolaryngologic Neoplasms] explode all trees<br><br>#3 MeSH descriptor: [Otorhinolaryngologic Neoplasms] explode all trees<br><br>#4 MeSH descriptor: [Head and Neck Neoplasms] explode all trees<br><br>#5 (head and neck):ti,ab,kw<br><br>#6 (larynx or laryngeal or glottis or glottic or oral cavity or oral or oropharynx or oropharyngeal or nasopharynx or nasopharyngeal or hypopharynx or hypopharyngeal or pharynx or pharyngeal or parapharyngeal or mouth or paranasal):ti,ab,kw	#1 "head and neck neoplasms"[MeSH Terms]<br><br>#2 "otorhinolaryngologic neoplasms"[MeSH Terms]<br><br>#3 "larynx"[Title/Abstract] OR "laryngeal"[Title/Abstract] OR "glottis"[Title/Abstract] OR "glottic"[Title/Abstract] OR "oral cavity"[Title/Abstract] OR "oral"[Title/Abstract] OR "oropharynx"[Title/Abstract] OR "oropharyngeal"[Title/Abstract] OR "nasopharynx"[Title/Abstract] OR "nasopharyngeal"[Title/Abstract] OR "hypopharynx"[Title/Abstract] OR "hypopharyngeal"[Title/Abstract] OR "pharynx"[Title/Abstract] OR "pharyngeal"[Title/Abstract] OR "parapharyngeal"[Title/Abstract] OR "mouth"[Title/Abstract] OR "paranasal"[Title/Abstract]<br><br>#4 "cancer*"[Title/Abstract] OR "carcinoma*"[Title/Abstract] OR "neoplasm*"[Title/Abstract] OR "tumor*"[Title/Abstract] OR "tumour*"[Title/Abstract] OR "metastas*"[Title/Abstract]<br><br>#5 "neoplasms"[MeSH Terms]	#1 ('head' OR 'head'/exp OR head) AND ('neck' OR 'neck'/exp OR neck) AND ('tumor' OR 'tumor'/exp OR tumor)<br><br>#2 ('head'/exp OR head) AND ('neck'/exp OR neck) AND ('neoplasms'/exp OR neoplasms)<br><br>#3 otorhinolaryngologic AND ('neoplasms'/exp OR neoplasms)<br><br>#4 'cancer*':ab,ti OR 'carcinoma*':ab,ti OR 'neoplasm*':ab,ti OR 'tumor*':ab,ti OR 'tumour*':ab,ti OR 'metastas*':ab,ti<br><br>#5 neoplasm<br><br>#6 #4 OR #5<br><br>#7 larynx:ti,ab,kw OR laryngeal:ti,ab,kw OR glottis:ti,ab,kw OR glottic:ti,ab,kw OR 'oral cavity':ti,ab,kw OR oral:ti,ab,kw OR oropharynx:ti,ab,kw OR oropharyngeal:ti,ab,kw OR nasopharynx:ti,ab,kw OR nasopharyngeal:ti,ab,kw

#7 #5 OR #6<br><br>#8 (cancer* or carcinoma* or neoplasm* or tumor* or tumour* or metastas*):ti,ab,kw<br><br>#9 MeSH descriptor: [Neoplasms] explode all trees<br><br>#10 #8 OR #9<br><br>#11 #7 AND #10<br><br>#12 #1 OR #2 OR #3 OR #4 OR #11<br><br>#13 (albumin to globulin ratio):ti,ab,kw<br><br>#14 (albumin globulin ratio):ti,ab,kw<br><br>#15 #13 OR #14<br><br>#16 #15 AND #12	#6 #4 OR #5<br><br>#7 #6 AND #3<br><br>#8 #1 OR #2 OR #7<br><br>#9 "Albumin/globulin ratio"[Title/Abstract]<br><br>#10 "albumin to globulin ratio" [Title/Abstract]<br><br>#11 "albumin-to-globulin ratio" [Title/Abstract]<br><br>#12 "albumin globulin ratio" [Title/Abstract]<br><br>#13 #9 OR #10 OR #11 OR #12<br><br>#14 #8 AND #13	OR hypopharynx:ti,ab,kw OR hypopharyngeal:ti,ab,kw OR pharynx:ti,ab,kw OR pharyngeal:ti,ab,kw OR parapharyngeal:ti,ab,kw OR mouth:ti,ab,kw OR paransal:ti,ab,kw<br><br>#8 #7 AND #6<br><br>#9 #1 OR #2 OR #3 OR #8<br><br>#10 ' Albumin/globulin ratio ':ti,ab,kw<br><br>#11 ' albumin to globulin ratio ':ti,ab,kw<br><br>#12' albumin-to-globulin ratio ':ti,ab,kw<br><br>#13' albumin globulin ratio ':ti,ab,kw<br><br>#14 #10 OR #11 OR #12 OR #13<br><br>#15 #9 AND #14

Table S2. Indices of methodological quality of the studies according to the Newcastle–Ottawa Quality Assessment Scale for cohort studies.

	**Selection**				**Comparability**	**Outcome**			
**Studies**	Representativeness of the exposed cohort	Selection of the non-exposed cohort	Ascertainment of exposure	Demonstration that outcome of interest was not present at start of study	Comparability of cohorts based on the design or analysis	Assessment of outcome	Was follow-up long enough for outcomes to occur	Adequacy of follow up of cohorts	**Total scores (stars)**
Du (2014)	*	*	*	*	*	*	*	-	7
Li (2015)	*	*	*	*	*	*	*	*	8
Chen (2017)	*	*	*	*	*	*	*	*	8
Gundog (2019)	*	*	*	*	*	*	*	*	8
Zhou (2019)	*	*	*	*	*	*	*	-	7
Zeng (2020)	*	*	*	*	*	*	-	-	6
Wang (2020)	*	*	*	*	*	*	*	*	8
Zhou (2020)	*	*	*	*	*	*	*	-	7
Zhuang (2021)	*	*	*	*	*	*	*	*	8
